# Effect of COVID-19 on the Generation of Waste in Marrakech, Morocco

**DOI:** 10.5696/2156-9614-11.30.210606

**Published:** 2021-05-28

**Authors:** Abdellah Ouigmane, Otmane Boudouch, Aziz Hasib, Omar Ouhsine, Elhoucein Layati, Rima J. Isaifan, Elhousseine Alaatchane, Ahmad Mottassadik, Mohamed Berkani

**Affiliations:** 1 Faculty of Sciences and Technics, University of Sultan Moulay Slimane, Beni Mellal, Morocco; 2 Laboratory of Landscape Dynamics, Risks and Heritage, University of Sultan of Moulay Slimane, Beni Mellal, Morocco; 3 Division of Sustainable Development, Hamad Bin Khalifa University, Qatar Foundation, Education City, Doha, Qatar; 4 Faculty of Letters and Human Sciences - Cadi Ayad University, Marrakech, Morocco; 5 Department of Physics, Cadi Ayyad University, Marrakech, Morocco

**Keywords:** COVID-19, household waste, construction and demolition waste, green waste, Morocco

## Abstract

**Background.:**

The production of solid waste continues to increase as the population and standard of living increases. In addition, changes in living conditions can induce significant variation in the quantitative and qualitative characteristics of waste.

**Objectives.:**

The aim of the present study was to examine the impact of the lockdown period on the generation of solid waste produced in the city of Marrakech.

**Methods.:**

The tonnage of household waste, construction and demolition waste and green waste was collected from the landfill and an analysis was made during the lockdown period in 2020 in comparison with the same period in 2019.

**Results.:**

The analysis of solid waste tonnage in 2019 and 2020 showed that the lockdown had a significant impact on the various wastes; with a 27.61% decrease for household waste, 6.27% decrease in the case of green waste, and 57.40% decrease for construction and demolition waste.

**Discussion.:**

The degree to which the tonnage of household waste decreased depended on the standard of living in each district which was defined by housing type. The tonnage of construction and demolition waste was influenced by the halt in construction activity in the city.

**Conclusions.:**

The results of the present study showed that the tonnage of household waste and demolition and construction waste decreased during the lockdown period.

**Competing Interests.:**

The authors declare no competing financial interests.

## Introduction

The daily production of household waste continues to increase globally due to population growth and improved living standards. According to the Word Bank, global household waste generation is expected to increase from 2.01 billion tons in 2016 to 3.40 billion tons by 2050.^1^

This evolution can be explained by improvements in living standards, population dynamics and the socio-economic context of each country.^2^,^3^ Studies conducted recently in Morocco have shown the impact of such changes on the production of household waste.^4^,^5^

In urban areas, the main wastes generated are household waste (HW), green waste (GW) and construction and demolition waste (CDW). The generation of HW depends on several parameters related mainly to the population and standard of living.^6^,^7^ Waste is defined in the Moroccan law as any waste resulting from economic, commercial, or artisanal activities (waste from house, restaurants, hotels, etc.). Green waste is defined as organic matter from agricultural waste (Law 28-00).^8^ The main sources of these wastes are public and villa gardens. Green waste is biodegradable, which favors its valorization in compost or methane production.^9^,^10^,^11^,^12^ Conversely, construction and demolition waste causes several issues for municipalities because of its weight. Construction and demolition waste does not present any physical or chemical interaction, and belongs to the class of inert waste (Law 28-00).^8^ This type of waste results from global urbanization,^13^ with an urbanization rate of 54.3% in 2016^14^,^15^ and 55% in 2018.^16^ To the best of our knowledge, there have been no studies on the quantitative characterization of GW and CDW in low- and middle-income countries.

Pandemics and global crises can have an impact on waste generation. Waste management becomes a major challenge for most countries in a pandemic due to changes in qualitative and quantitative aspects of generated waste.^4^ Two to three months after its appearance in Wuhan, China on 17 November 2019, COVID-19 became a global pandemic.^17^,^18^,^19^,^20^,^21^ Morocco, like other countries, was affected by the pandemic; with the first case detected on 2 March 2020. By 27 June 2020, the country had registered 11854 confirmed cases including 8700 recovered and 218 deaths.^22^ Morocco is among the countries that applied the measures of public health confinement as early as 20 March 2020 when the Moroccan government declared a state of public health emergency and went into mandatory lockdown except for emergencies.^23^ COVID-19 was first identified in the city of Marrakech on 9 March 2020. By 27 June, 1426 cases were confirmed, including 1259 who had recovered and 45 deaths.^22^

**Abbreviations**
*CDW*: Construction and demolition waste
*GW*: Green waste
*HW*: Household waste

The present study was conducted to assess the impact of COVID-19 on solid waste generation in the city of Marrakech, Morocco. The types of waste included household waste, green waste and construction and demolition waste.

## Methods

Marrakech is located in central Morocco with an estimated population of 914000 inhabitants in 2020. The city covers an area of 230 km^2^. It is the fourth largest city in Morocco and is subdivided into six districts.

### Waste management in the study area

**Figure 1:**
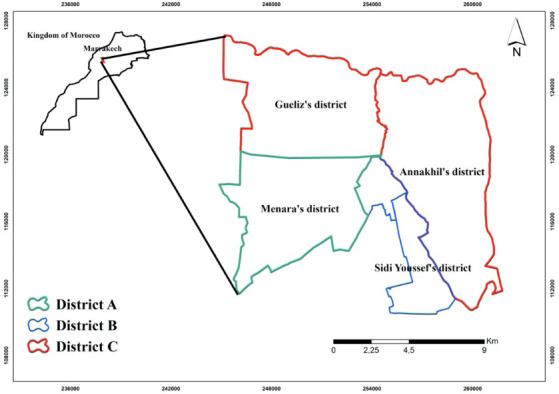
Division of the city of Marrakech according to waste collection

In 2014, the districts of the city of Marrakech signed a waste collection contract with three private companies. The areas of operation for these companies are shown in Figure 1. Before 2019, the collected waste was buried in Marrakech's public landfill. Before 2019, the city of Marrakech buried waste in an uncontrolled landfill. Because of the impacts generally related to the pollution of groundwater by leachate, the lack of control of biogas emissions and the health impacts on ragpickers, the municipality decided, in partnership with the Minister of the Environment, to build a new landfill and recovery center to treat the leachate and biogas and recover 50% of the waste sent to the landfill (composting, refuse derived fuel (RDF) production and recycling). A 20-year contract was signed with a private operator.^24^

### Production of waste in the study area

The objective of the present study was to perform a quantitative analysis of waste generation before and during the COVID-19 lockdown (three months from March 2020 to May 2020). Table 1 presents data on population and estimated tonnage of waste in each district calculated using Equation 1.

**Table 1:** Population and Municipal Solid Waste (MSW) Generation in the City of Marrakech

**Symbol of district**	**Name of district**	**Population in 2019^26^**	**Rate of household waste production (kg per capita per day)**	Daily estimated production of MSW (tons/day)*
District A	Gueliz + Annakhil	25,7552	0.76	197
District B	Medina + Sidi Youssef Ben ali	243,330	0.76	185
District C	Menara	411,897	0.76	314

			**Total**	**696**

^*^ Daily estimated production of MSW (Tons/day) = Population ^*^ rate of waste production (kg per capita per day)/1000


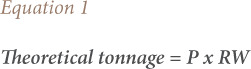


Where *RW* is the rate of household waste production in urban areas in Morocco (0.76 kg per capita per day)^25^ and P is the estimated population in each district.^26^

Actual tonnage data collected from the landfill was used to observe the impact of lockdown. Before each collection, trucks enter the landfill to empty the waste, the type of waste is then identified (HW, GW, or CDW). The trucks then pass over a weighbridge to determine the net tonnage of the waste. Data on waste tonnage were recovered from the landfill for analysis in the present study.

## Results

Quantitative analysis of differences in waste tonnage between the three months (March, April and May) of 2019 and the corresponding period during lockdown in 2020 showed that lockdown had a noticeable effect on the generation of household waste. Figure 3 shows the change in tonnage in HW production from district A. The quantity of this category of waste decreased during the three months corresponding to the lockdown period. Similarly, Figure 3 and 4 present the changes in the tonnage in HW produced in district B and C, respectively. The impact on the daily waste generation ratio is presented in Supplemental Material Table 1.

### Green waste

The production of green waste concerned only districts A and C due to the presence of public gardens and villas. As shown in Figures 5 and 6, the lockdown did not influence the production of this category of waste, except for the month of May, which reported on a decrease in the tonnage of waste produced from both districts during the lockdown period.

### Construction and demolition waste

As shown in Figures 7, 8 and 9, the tonnage of demolition and construction waste decreased significantly in all months of the year in 2020. The only exception was the month of March in District A where the tonnage of CDW increased. The impact on the daily waste generation ratio is presented in Supplemental Material Table 2.

## Discussion

**Figure 2:**
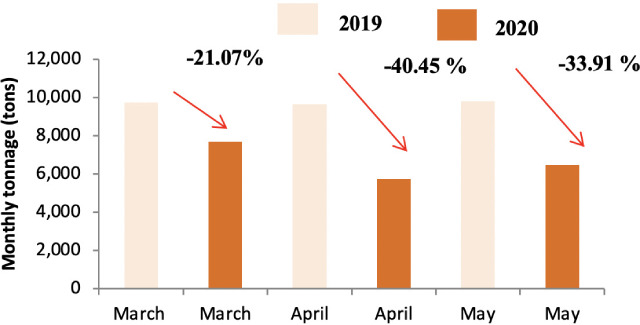
Comparison of HW tonnage in 2019 and 2020 in district A

The highest decrease in waste produced was reported for April for district A and B with a decrease of around 40% and the highest decrease in waste produced in district C was found in May with a decrease of around 22%. In addition, the results showed that the lockdown period contributed to a decrease in the daily production ratio for all districts; from 1.23 to 0.84 kg per capita per day for District A, from 0.92 to 0.60 kg per capita per day for District B, and from 0.88 to 0.73 kg per capita per day for District C *(Supplemental Material Table 1).* These results are in agreement with a recent report^4^ on the effect of lockdown on the tonnage of household waste in a small town and village in Morocco which showed that the rate of decrease depended on the typology of the area (urban versus rural). A study was conducted in the same field to evaluate the impact of COVID-19 on waste generation in three different regions. The results showed a decrease in the quantities of waste generated in the city of Shanghai (−34%) during the lockdown period, on the other hand the tonnage increased in Singapore (+3%) and Brno in the Czech Republic (+1%).^27^ In the present study, the percentage decrease varied across the different districts. There was less of a decrease in district C compared to districts A and B. Indeed, the standard of living in district B was low compared to the other districts; this decrease in waste production was mainly related to the change in consumption patterns due to the effects of the pandemic. This district is home to Jamaa Lafna, which is a popular tourist attraction. The abrupt interruption of national and international traffic due to the COVID-19 crisis resulted in a sudden halt in activity, with a significant loss of income in terms of employment and contribution to the economy in general, especially for tourist cities such as Marrakech.^28^ During the lockdown, tourist activities were banned and hence a decrease in produced waste tonnage was observed in this area. The same was noted for District A, where the decrease in waste tonnage was caused by the closure of a large number of tourist complexes that characterize this district *(Figure 2).* The lower decrease waste tonnage in the month of March compared to other months is due to the start date of the containment which was announced around 20 March 2020. A possible explanation for decreased waste generation might be reduced food intake during the pandemic.^29^

**Figure 3:**
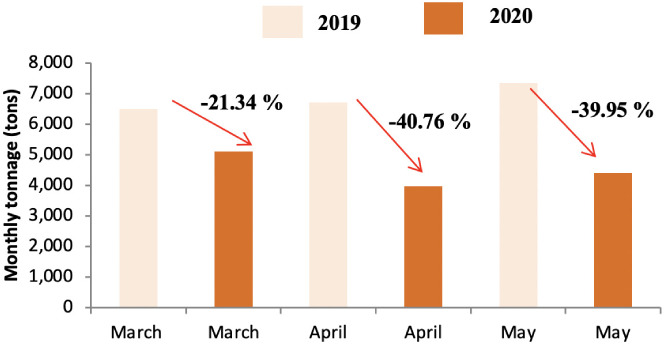
Comparison of household waste tonnage in 2019 and 2020 in district B

**Figure 4:**
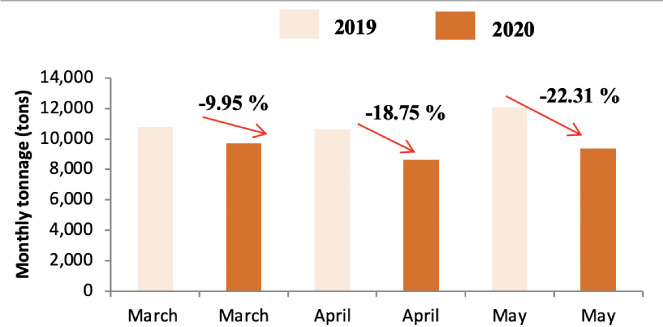
Comparison of household waste tonnage in 2019 and 2020 in district C

**Figure 5:**
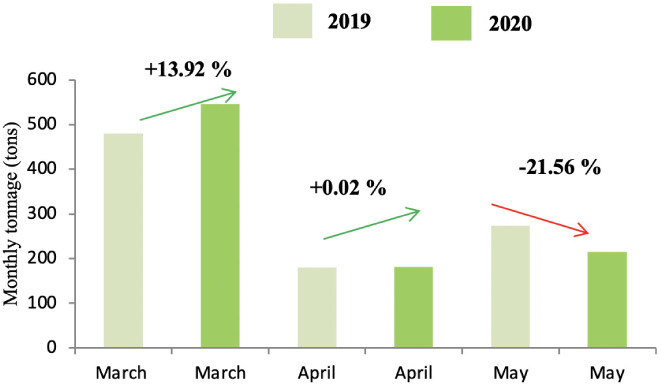
Comparison of green waste tonnage in 2019 and 2020 in district A

**Figure 6:**
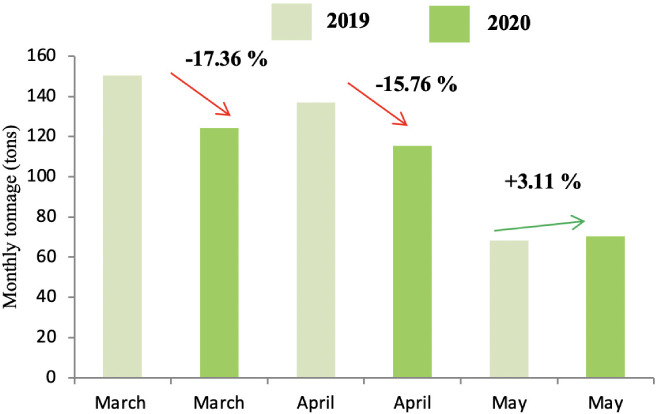
Comparison of green waste tonnage in 2019 and 2020 in district C

**Figure 7:**
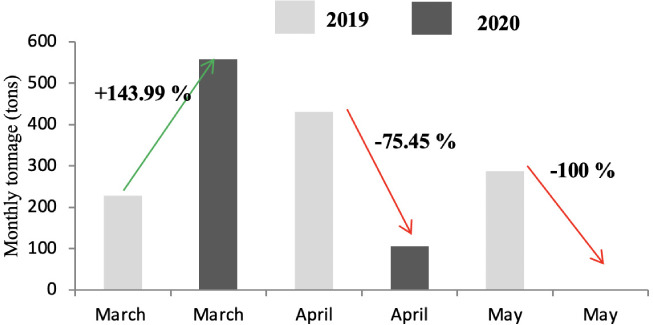
Comparison of construction and demolition waste tonnage in 2019 and 2020 in district A

**Figure 8:**
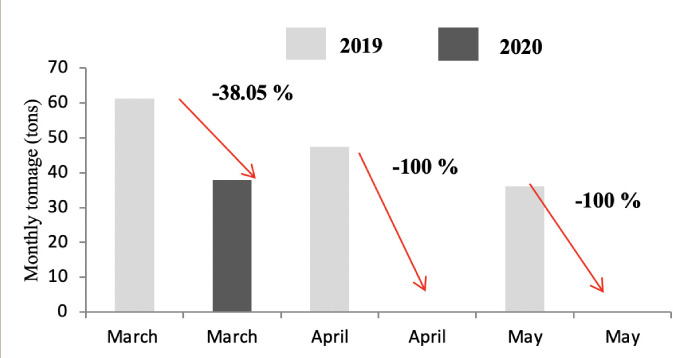
Comparison of construction and demolition waste tonnage in 2019 and 2020 in district B

**Figure 9:**
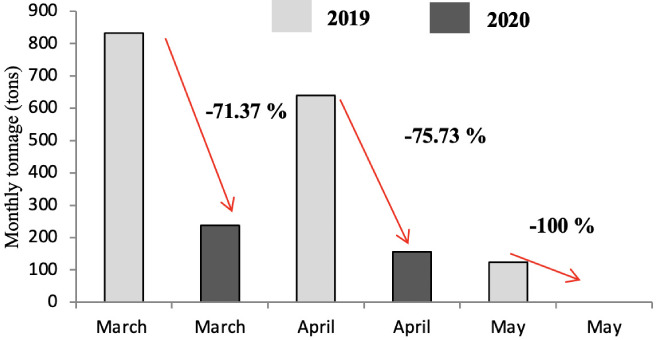
Comparison of construction and demolition waste tonnage in 2019 and 2020 in district C

### Green waste

Green waste is generated from the maintenance of public gardens and villa areas. This type of waste is not produced in district B as it does not contain many villas. On the other hand, GW is still produced in districts A and C *(Figures 5 and 6),* with a high production rate during the months of March and April (corresponding to the spring months). Moreover, the lockdown did not show any influence on the production of this waste in district A because the maintenance of gardens in these areas did not stop during the confinement. In addition to the presence of public gardens, the standard of living in the district is high and it is home to a large number of villas, which explains the high quantities of GW in this district. The drop in the evolution of GW in district C *(Figure 6)* can be explained by the decrease in the frequency of maintenance of public gardens.

### Construction and demolition waste

The production of CDW is an issue for the majority of municipalities. The management of this type of waste is difficult, especially in the collection phase, because of its heavy weight. The generation of CDW stems from urban construction activities. Marrakech is a large Moroccan city with a significant urban sprawl, which generates large quantities of gravel. The amount of CDW generated is higher in Districts A and C because of the more frequent construction activity in these areas compared to district B, which is characterized by limited space. As seen in Figures 7, 8 and 9 and Supplemental Material Table 2, lockdown had a significant influence on the generation of this type of waste. In contrast to the month of May 2019, in the same period in 2020 no rubble production was recorded in the three districts of the city. The drop in CDW generation was due to the halt in construction work because of the economic and psychological effects of the pandemic on the population.^30^,^31^

## Conclusions

The present study examined the impact of lockdown on the generation of household waste, green waste and construction and demolition waste. The results of the analysis showed that lockdown had a significant impact of on the generation of HW, as waste tonnage decreased during the confinement period compared to the same period in 2019. Similarly, the production of CDW decreased during the lockdown period. On the other hand, lockdown had less of an impact on the production of GW due to the continuity of garden maintenance despite confinement measures.

In addition to the benefits of lockdown on air quality and ecosystems, the present study showed that lockdown resulted in a drop in the tonnage of waste produced which can have potential benefits for the environment. In the other hand, the period of the pandemic may have a negative impact on the composition of household waste. Companies managing the landfills were affected by the decrease in tonnage as they are paid on a per tonnage basis.

The conditions for the present study were unique, as it was conducted during a lockdown which lasted a period of three months. Further research is needed to determine the main parameters influencing the qualitative aspects of waste during pandemics over a longer period of time.
