# Supplementary material for: Effect of COVID-19 on the Generation of Waste in Marrakech, Morocco
Source: J Health Pollut. 2021 May 28;11(30):210606. doi: 10.5696/2156-9614-11.30.210606 (PMC8276732; doi:10.5696/2156-9614-11.30.210606)
Supplement: Supplementary file 1 [file Ouigmane_Supplemental_Material.docx]

**Supplemental Material**

Supplemental Material Table 1: Impact of Lockdown on Household Waste Production Rate

|  | **2019** | | | | | | | **2020** | | | | | | |
| --- | --- | --- | --- | --- | --- | --- | --- | --- | --- | --- | --- | --- | --- | --- |
|  | Population | Tonnage March | Tonnage April | Tonnage May | Average (ton per month) | Rate of HW production (kg/inhabitant/day)* | Population | | Tonnage March | Tonnage April | Tonnage May | Average (ton per month) | Rate of HW production (kg/inhabitant/day) |  |
| District A | 257552 | 9749 | 9645 | 9793 | 9729 | 1.23 | 257589 | | 7695 | 5744 | 6471 | 19910 | 0.84 |  |
| District B | 243330 | 6491 | 6698 | 7342 | 6844 | 0.92 | 243290 | | 5106 | 3968 | 4409 | 13483 | 0.60 |  |
| District C | 411897 | 10779 | 10638 | 12087 | 11168 | 0.88 | 412058 | | 9706 | 8644 | 9390 | 27740 | 0.73 |  |

Abbreviation: HW, household waste production

* The rate of HW production is calculated by dividing the average monthly tonnage by the population multiplied by 1000.

**Supplemental Material Table 2:** Impact of Lockdown on Construction and Demolition Waste Production Rate

|  | **2019** | | | | | | **2020** | | | | | |
| --- | --- | --- | --- | --- | --- | --- | --- | --- | --- | --- | --- | --- |
|  | Population | Tonnage March | Tonnage April | Tonnage May | Average (ton per month) | Rate of CDW production (kg/inhabitant/day) | Population | Tonnage March | Tonnage April | Tonnage May | Average (ton per month) | Rate of CDW production (kg/inhabitant/day) |
| District A | 257552 | 229 | 431 | 287 | 316 | 3.68 | 257589 | 558 | 106 | 0 | 221 | 2.58 |
| District B | 243330 | 61 | 47 | 36 | 48 | 0.59 | 243290 | 38 | 0 | 0 | 13 | 0.16 |
| District C | 411897 | 832 | 640 | 124 | 532 | 3.87 | 412058 | 238 | 155 | 0 | 131 | 0.95 |
